# Numerical analysis & modelling in the life sciences: a topical collection in honor of Ezio Venturino

**DOI:** 10.1007/s00285-026-02388-0

**Published:** 2026-05-13

**Authors:** Iulia Martina Bulai, Roberto Cavoretto, Alessandra De Rossi

**Affiliations:** Department of Mathematics Giuseppe Peano, University of Torino, Via Carlo Alberto 10, 10123 Torino, Italy

**Keywords:** Mathematical biology, Mathematical modelling, Numerical analysis, Population dynamics, Eco-epidemiological models, 92-XX

## Abstract

This topical collection of the *Journal of Mathematical Biology* is dedicated to Professor Ezio Venturino on the occasion of his 70th birthday. It brings together recent advances in the mathematical modelling and analysis of biological systems, spanning topics from dynamical systems and stochastic processes to applications in ecology, epidemiology, evolutionary dynamics, and biomedicine. The contributions highlight both new theoretical developments and innovative computational techniques, reflecting the vitality of current research in mathematical biology and offering a fitting tribute to Professor Venturino’s influential scientific career.

## Introduction

The interface between mathematics and the life sciences has long been a productive field for advancing both disciplines. Mathematical models, supported by rigorous analysis and computational techniques, allow us to gain a deeper understanding of the dynamics of biological systems, to generate predictions, and to provide guidance in addressing complex problems in ecology, epidemiology, evolution, and medicine.

This topical collection of the *Journal of Mathematical Biology*, entitled *Numerical Analysis & Modelling in the Life Sciences*, is dedicated to Professor Ezio Venturino on the occasion of his 70th birthday. Professor Venturino made influential contributions across a broad spectrum of mathematical biology, with particular impact in the fields of mathematical ecology, population dynamics, and the analysis of interactions in complex biological systems. Among his many contributions are influential studies on predator–prey and eco-epidemiological systems, which helped clarify how infectious diseases interact with ecological dynamics and population persistence (Venturino 1994, 2001, 2002; Petrovskii et al. 2002; Malchow et al. 2007; Haque et al. 2011; Ajraldi et al. 2011; Bulai and Venturino 2017; Acotto and Venturino 2023). His scientific legacy, Acotto et al. (2025), together with his commitment to fostering collaboration between mathematics and the life sciences, continues to inspire colleagues, collaborators, and students around the world.

Professor Ezio Venturino has had a long and distinguished career in applied mathematics. After completing his Master’s degree in Mathematics at the University of Turin, he obtained a Ph.D. in Applied Mathematics at the State University of New York at Stony Brook. He subsequently held research and academic positions at several institutions, including the University of Iowa, the University of Catania, and the Polytechnic University of Turin, before becoming Professor of Numerical Analysis at the University of Turin. Over the course of his career he has combined work in numerical analysis, dynamical systems, and mathematical modelling, with a strong emphasis on interdisciplinary applications in the life sciences.

A large part of Venturino’s research has focused on mathematical ecology and epidemiology. His work has contributed to the development of eco-epidemiological models, which study the interplay between ecological interactions and the spread of infectious diseases within populations. In addition to numerous studies on predator–prey systems, population dynamics, and disease transmission, he co-authored with H. Malchow and S. Petrovskii a widely known monograph on spatiotemporal patterns in ecology and epidemiology. Through more than three hundred publications, Venturino has combined rigorous analysis of nonlinear dynamical systems with biologically motivated modelling, helping to strengthen the role of mathematical approaches in understanding ecological and epidemiological processes.

This collection is also closely connected to the themes of the International Conference *Numerical Analysis & Modelling in Applied Sciences (NAMAS-24)*, held in Gaeta, Italy, in September 2024, which brought together researchers working at the intersection of mathematics and the applied sciences.

## Overview of the contributions

The ten contributions published in this collection reflect the scope and vitality of ongoing advances in mathematical biology, a field to which Professor Venturino devoted much of his career. They present new analytical methods, innovative computational approaches, and novel applications that advance our understanding of biological phenomena. The articles span diverse topics, including dynamical systems, stochastic processes, epidemiological and ecological models, population and evolutionary dynamics, and Alzheimer modelling. Collectively, they illustrate how mathematical and computational perspectives can shed light on fundamental questions in the life sciences while opening new avenues for interdisciplinary research. In the following, we briefly summarize the contributions to this Special Collection.

### Positive and negative roles of random perturbations in a tumor–immune system with treatment

This contribution investigates how random perturbations influence tumor–immune dynamics under chemotherapy, Bashkirtseva and Ryashko (2025a). Bashkirtseva and Ryashko study a mathematical model describing the interaction between tumor cells and immune effector cells in the presence of treatment, focusing on how stochastic effects influence therapeutic outcomes. For the deterministic formulation of the model, regions of mono-, bi-, and tristability are identified as functions of the treatment intensity, revealing equilibrium and oscillatory attractors associated with active, dormant, and tumor-free states. Building on this bifurcation analysis, the stochastic version of the system is investigated.

### The role of random perturbations in the dynamic variability of a discrete predator-prey model: a stochastic sensitivity analysis

Bashkirtseva and Ryashko investigate the role of stochastic fluctuations in a discrete predator-prey model with Holling type II functional response, Bashkirtseva and Ryashko (2025b). Even in the deterministic setting, the model displays complex dynamics including bistability and chaotic regimes. By employing stochastic sensitivity techniques and confidence domain methods, the authors analyze how random perturbations drive transitions between persistence and extinction, giving insight into the geometric and dynamical mechanisms underlying noise-induced phenomena in population dynamics.

### Optimal control of monomers and oligomers degradation in an Alzheimer’s disease model

Bulai, Ferraresso, and Gladiali propose a new model for Alzheimer’s disease progression based on the interactions of monomers, proto-oligomers, and oligomers of amyloid proteins, Bulai et al. (2025). The model captures key biochemical processes and is extended with optimal control strategies targeting monomers and/or oligomers. Numerical simulations show that combined treatments yield the most effective reduction of toxic oligomers, while targeting oligomers alone outperforms monomer-specific interventions. These findings provide mathematical support for immunotherapy strategies against Alzheimer’s disease.

### Modelling behavioural changes and vaccination in the transmission of respiratory viruses with co–infection

Another paper addresses the interplay between human behaviour, vaccination, and the transmission of two respiratory viruses with possible co-infection, Buonomo and Penitente (2025). Buonomo and Penitente incorporate information-driven behavioural changes and study their impact through bifurcation analysis, extending the framework to include vaccination strategies and seasonal effects. Their results reveal how information, rumours, and reduced vaccination coverage can amplify epidemic peaks, emphasizing the importance of integrating behavioural dynamics into epidemiological modelling.

### A replicator model with transport dynamics on networks for species evolution

In this contribution, Coclite, Pellegrino, Politi and Popolizio, introduce a multi-scale framework for studying species evolution in marine ecosystems, Coclite et al. (2025). Local evolutionary processes within reserves are modeled by the replicator equation, while inter-reserve connectivity is represented through transport dynamics on networks. The analysis demonstrates how equilibria and stability properties depend on network structure and exchange rates, and numerical experiments validate the proposed high-order integration scheme. The results highlight the model’s potential for capturing synchronization phenomena and providing insights into ecosystem resilience and management.

### Analytical and numerical properties of an extended angiogenesis PDE model

De Luca and Marcellino develop an extended partial differential equation model for tumor angiogenesis in which oxygen dynamics play a key regulatory role, De Luca and Marcellino (2025). Building on a five-component framework describing endothelial cells, proteases, inhibitors, extracellular matrix, and oxygen concentration, the authors establish existence, uniqueness, and boundedness of solutions and introduce a numerical scheme based on the method of lines and a fourth-order Runge–Kutta integrator with proven stability and convergence properties. Numerical simulations reproduce biologically plausible angiogenic patterns and highlight the influence of oxygen in shaping vascular dynamics.

### Studying the role of phenotypic change in biological invasion success through mathematical modeling

Another contribution investigates the role of phenotypic plasticity and adaptation in the success or failure of biological invasions, Rivera-Estay et al. (2025). Rivera-Estay, Moreno-Gómez, Córdova-Lepe, Gutiérrez and Benítez propose an eco-evolutionary predator-prey model involving a native predator-prey system exposed to an exotic invader. By analyzing inducible defenses in prey and inducible offenses in predators, they identify the conditions under which invasion succeeds or fails. Numerical experiments, inspired by the invasive dynamics of the American mink, demonstrate how efficiency, costs, and the relative speed of phenotypic change govern invasion outcomes.

### Multi-Compartmental Staged Progression Endemic Models with Fast Transitions

Sanz-Lorenzo, Bravo de la Parra, Poggiale and Auger introduce a multi-compartmental staged progression epidemic model in which individuals transition between compartments on a faster time scale, Sanz-Lorenzo et al. (2025). By reducing the full system, the authors characterize disease eradication and endemicity through the basic reproduction number, establishing links between global outcomes and compartment-specific dynamics. The analysis shows how transition rates determine whether endemicity or eradication prevails, offering theoretical insights and potential tools for managing epidemics in structured populations.

### Bistability and complex bifurcation diagrams generated by waning and boosting of immunity

In this contribution, Scarabel, Polner, Wylde, Barbarossa and Röst, analyze an epidemiological model with waning immunity and boosting upon re-exposure, formulated as an SIRS-type system with discrete and distributed delays, Scarabel et al. (2025). Using advanced numerical continuation methods, the authors uncover a rich bifurcation structure including catastrophic bifurcations, torus bifurcations, and bistability of periodic solutions. Their results extend earlier analyses and highlight the potential long-term public health consequences of interventions that shift the system between coexisting attractors.

### Numerical bifurcation analysis of turing and symmetry broken patterns of a PDE model for vegetation dynamics

In the final contribution, Spiliotis, Russo, Siettos, and Giannino study pattern formation in vegetation dynamics in water-limited environments, Spiliotis et al. (n.d.). The model consists of two reaction–diffusion equations for biomass and water coupled with an ordinary differential equation describing biomass-dependent toxicity. Through linear stability analysis in a one-dimensional bounded domain, the authors derive analytical conditions for the onset of Turing instability and characterize their dependence on the domain size. A complementary numerical bifurcation analysis with respect to the precipitation rate reveals the structure and stability of spatially inhomogeneous solutions, uncovering secondary bifurcations and symmetry-breaking patterns that lead to multistability phenomena.

Together, these ten contributions illustrate the strength and diversity of approaches in contemporary mathematical biology, from theoretical developments in dynamical systems and stochastic analysis to applied models addressing important biomedical and epidemiological challenges.

## Concluding remarks

We are confident that this topical collection will serve both as a valuable resource for the mathematical biology community and as a fitting tribute to Professor Venturino’s scientific achievements and his sustained commitment to interdisciplinary collaboration. The authors sincerely thank Professor Ezio Venturino for his lasting contributions to applied mathematics and for promoting fruitful dialogue between mathematics and the life sciences.

## Data Availability

Data sharing is not applicable to this article as no new data were created or analyzed in this study.
